# Understanding the effect of an educational intervention to optimize HIV testing strategies in primary care in Amsterdam – results of a mixed-methods study

**DOI:** 10.1186/s12875-023-02161-y

**Published:** 2023-09-30

**Authors:** Saskia Bogers, Pythia Nieuwkerk, Nynke van Dijk, Maarten Schim van der Loeff, Suzanne Geerlings, Jan van Bergen, the HIV Transmission Elimination Amsterdam (H-TEAM) Consortium, T. van Benthem, D. Bons, G. J. de Bree, P. Brokx, U. Davidovich, F. Deug, M. Heidenrijk, E. Hoornenborg, M. Prins, P. Reiss, A. van Sighem, M. van der Valk, J. de Wit, W. Zuilhof, N. Schat, D. Smith, M. van Agtmael, J. Ananworanich, D. van de Beek, G. E. L. van den Berk, D. Bezemer, A. van Bijnen, J. P. Bil, W. L. Blok, M. Bomers, A. Boyd, W. Brokking, D. Burger, K. Brinkman, M. de Bruin, S. Bruisten, L. Coyer, R. van Crevel, M. Dijkstra, Y. T. van Duijnhoven, A. van Eeden, L. Elsenburg, M. A. M. van den Elshout, E. Ersan, P. E. V. Felipa, T. B. H. Geijtenbeek, J. van Gool, A. Goorhuis, M. Groot, C. A. Hankins, A. Heijnen, M. M. J. Hillebregt, M. Hommenga, J. W. Hovius, N. Brinkman, Y. Janssen, K. de Jong, V. Jongen, N. A. Kootstra, R. A. Koup, F. P. Kroon, T. J. W. van de Laar, F. Lauw, M. M. van Leeuwen, K. Lettinga, I. Linde, D. S. E. Loomans, I. M. van der Lubben, J. T. van der Meer, T. Mouhebati, B. J. Mulder, J. Mulder, F. J. Nellen, A. Nijsters, H. Nobel, E. L. M. Op de Coul, E. Peters, I. S. Peters, T. van der Poll, O. Ratmann, C. Rokx, W. E. M. Schouten, J. Schouten, J. Veenstra, A. Verbon, F. Verdult, J. de Vocht, H. J. de Vries, S. Vrouenraets, M. van Vugt, W. J. Wiersinga, F. W. Wit, L. R. Woittiez, S. Zaheri, P. Zantkuijl, A. Żakowicz, M. C. van Zelm, H. M. L. Zimmermann

**Affiliations:** 1 Department of Internal Medicine, Amsterdam UMC Location University of Amsterdam, Meibergdreef 9, Amsterdam, The Netherlands; 2 Amsterdam Institute for Infection and Immunity, Infectious Diseases, Amsterdam, the Netherlands; 3 Amsterdam Public Health Research Institute, Quality of Care, Amsterdam, the Netherlands; 4 Department of Medical Psychology, Amsterdam UMC Location University of Amsterdam, Meibergdreef 9, Amsterdam, The Netherlands; 5 Amsterdam Public Health Research Institute, Mental Health, Personalized Medicine, Amsterdam, the Netherlands; 6 Department of General Practice, Amsterdam UMC Location University of Amsterdam, Meibergdreef 9, Amsterdam, The Netherlands; 7 Amsterdam University of Applied Sciences, Faculty of Health, Center of Expertise Urban Vitality Amsterdam, Amsterdam, The Netherlands; 8 Department of Infectious Diseases, Public Health Service of Amsterdam, Amsterdam, the Netherlands; 9 STI AIDS Netherlands, Amsterdam, the Netherlands

**Keywords:** Mixed-methods, HIV testing, General practitioner, Primary care, Medical education, Sexually transmitted infections

## Abstract

**Background:**

In the Netherlands, general practitioners (GPs) play a key role in provider-initiated HIV testing, but opportunities for timely diagnosis are regularly missed. We implemented an educational intervention to improve HIV testing by GPs from 2015 to 2020, and observed a 7% increase in testing in an evaluation using laboratory data. The objective for the current study was to gain a deeper understanding of whether and how practices and perceptions of GPs’ HIV/sexually transmitted infection (STI) testing behaviour changed following the intervention.

**Methods:**

We performed a mixed-methods study using questionnaires and semi-structured interviews to assess self-reported changes in HIV/STI testing by participating GPs. Questionnaires were completed by participants at the end of the final educational sessions from 2017 through 2020, and participating GPs were interviewed from January through March 2020. Questionnaire data were analysed descriptively, and open question responses were categorised thematically. Interview data were analysed following thematic analysis methods.

**Results:**

In total, 101/103 participants completed questionnaires. Of 65 participants that were included in analyses on the self-reported effect of the programme, forty-seven (72%) reported it had changed their HIV/STI testing, including improved STI consultations, adherence to the STI consultation guideline, more proactive HIV testing, and more extragenital STI testing. Patients’ risk factors, patients’ requests and costs were most important in selecting STI tests ordered. Eight participants were interviewed and 15 themes on improved testing were identified, including improved HIV risk-assessment, more proactive testing for HIV/STI, more focus on HIV indicator conditions and extragenital STI testing, and tools to address HIV during consultations. However, several persistent barriers for optimal HIV/STI testing by GPs were identified, including HIV-related stigma and low perceived risk.

**Conclusions:**

Most GPs reported improved HIV/STI knowledge, attitude and testing, but there was a discrepancy between reported changes in HIV testing and observed increases using laboratory data. Our findings highlight challenges in implementation of effective interventions, and in their evaluation. Lessons learned from this intervention may inform follow-up initiatives to keep GPs actively engaged in HIV testing and care, on our way to zero new HIV infections.

**Supplementary Information:**

The online version contains supplementary material available at 10.1186/s12875-023-02161-y.

## Introduction

HIV transmission remains an important public health issue, with 106,508 people newly diagnosed with HIV in the European Region in 2021 [1]. As transmission is prevented through adequate therapy, most transmissions come from persons with undiagnosed HIV [2, 3]. Optimal HIV testing strategies are therefore crucial to end the HIV epidemic.

In the Netherlands, general practitioners (GPs) perform two thirds of sexually transmitted infections (STI) consultations and diagnose one third of HIV infections [4]. GPs therefore play a crucial role in provider-initiated HIV testing, in particular among people not attending sexual health centres (SHCs) [5]. They are advised to offer HIV testing to patients who have a significant HIV risk according to their history, to patients presenting with symptoms suggestive of an acute HIV infection, and to patients presenting with HIV indicator conditions such as STIs, mononucleosis-like illness, unexplained weight-loss, diarrhoea or fever, herpes zoster, and community acquired pneumonia. GP’s are additionally advised to periodically offer HIV testing to high-risk patients, and all patients when they hold practice in a high-prevalence area (> 2 per 1000 persons) [6]. SHCs provide free-of-charge HIV testing on an opt-out basis for key groups only (i.e. people being notified for an STI, people having STI symptoms, men who have sex with men [MSM], people with a non-Western migratory background and people aged < 25 years); in contrast, HIV testing by GPs is not covered by health insurance if the obligatory annual deductible (currently €385) has not been reached [5].

Previous research indicated that opportunities for earlier HIV diagnosis are being missed in primary care [7]. Implementing optimal HIV testing practices in primary care is an ongoing challenge, especially in a low-prevalence setting and in the context of a shrinking epidemic [8]. In 2014, a consortium of stakeholders in HIV care launched the HIV Transmission Elimination in Amsterdam (H-TEAM) initiative, which aims to implement innovative interventions for improved HIV prevention, testing and care though a city-based approach. In such an approach, multilevel citywide initiatives are employed through existing municipal infrastructures, to ensure optimal impact [9]. From 2015 to 2020, the H-TEAM implemented an educational intervention programme aiming to improve HIV testing strategies by GPs as well as the quality of GPs’ STI consultations in general [10]. We previously reported the effect of the programme by assessing changes in HIV/STI testing frequencies by participating GPs compared to non-participating GPs as the primary outcome, using laboratory data. The intervention was associated with a 7% increase in HIV testing among participating GPs and has been described elsewhere [11]. Although an increase in HIV testing indeed was the primary objective of the intervention, the quality of HIV/STI testing by GPs can only partially be assessed from anonymised laboratory data as no information was available on risk factors and reasons for testing. To put the results from the laboratory-based evaluation in perspective, we used questionnaires and interviews with participating GPs to gain a deeper understanding of their practices and the perceptions of their testing behaviour. Additionally, we aimed to identify contextual factors influencing GPs’ HIV/STI testing behaviour that need to be addressed in the future.

## Methods

### Design and setting

We performed a mixed-methods study using questionnaires and semi-structured interviews among Amsterdam-based GPs who participated in the intervention. The educational intervention programme consisted of two consecutive small group sessions; all Amsterdam GPs were invited to participate. During the sessions, trends in incidence and prevalence of HIV/STI and current guidelines were discussed in existing groups of 5 to 20 GPs who regularly attend continuing medical education (CME) sessions together. Barriers to appropriate HIV/STI testing that were previously identified by our group were addressed during these discussions [12]. Graphical audit and feedback (i.e. assessment of an individual’s professional performance and comparison and graphical feedback of that performance to the individual and their peers) based on laboratory data was presented to discuss differences in test-ordering between participants as a stimulant to improving testing [13]. At the end of the first educational session (Session-I), participating GPs developed quality improvement plans for HIV/STI testing in their own practice, and their implementation was discussed in the second educational session (Session-II). Further details on the design and implementation of the programme are described elsewhere [10].

### Questionnaire recruitment and design

All GPs attending Session-II were invited to complete a questionnaire containing four sections: (1) participant characteristics, (2) perceived effect of the programme, (3) implementation of quality improvement plans, and (4) programme evaluation (Supplementary Table 1). The questionnaire was developed by a group of experts on HIV medicine, primary care and medical education, led by NvD and JvB and updated after piloting in the first two sessions.

### Interview recruitment and design

GPs who participated in both educational sessions were eligible to participate in semi-structured interviews, and were invited by email. No additional selection based on GPs’ characteristics was applied. GPs were invited until data saturation was achieved. The interviews were structured by a topic guide, consisting of open-ended questions on (1) effect of the programme, (2) reflection on trends in HIV/STI testing by Amsterdam GPs, (3) experienced barriers and facilitators to HIV/STI testing, and (4) programme evaluation (Supplementary Table 2). The topic guide was developed by experts in the field of medical psychology, primary care, infectious diseases, HIV medicine, and medical education (PN, NvD, JvB, SG, MSvdL and SJB). After four interviews, the topic guide was reviewed and updated based on interim analyses.

### Data collection

The questionnaire was completed on paper at each Session-II (2017–2020), and data were entered into Castor Electronic Data Capture. Interview data were collected through individual interviews conducted by SJB between January and March 2020. The duration of the interviews ranged from 25 to 54 min. Interviews took place at a location of choice of the participant or by telephone, and were audio recorded. Interviews were transcribed verbatim and data were anonymised. As data collection was completed before the start of the COVID-19 pandemic (March 2020), no adjustments to correct for any effect this may have had on our findings were made.

### Analysis

All questionnaires completed by GPs were included for analysis, regardless of missing items. For items on the programme’s effect on HIV/STI testing behaviour, only responses from GPs who participated in both sessions were included, as those who only attended Session-II could not yet report on changes in testing behaviour. Data on participant characteristics, implementation of the quality improvement plans and the programme’s evaluation were analysed descriptively. Open question responses were categorised thematically (SJB), and checked for agreement (MSvdL). All questionnaire data analyses were performed using Stata v15.1 (StataCorp LLC, College Station, Texas, USA).

Interview transcripts were analysed by two independent researchers (SJB and PN) following reflexive thematic analysis methods by Braun and Clarke [14]. The researchers started an open coding process using the first three interviews, which resulted in a preliminary code system through consensus discussion that was built upon. The final categorization of identified themes was reached through consensus discussion. Interview data were analysed using MaxQDA 2022 (VERBI Software, Berlin, Germany).

## Results

### Participant characteristics

Overall, 36% (229/632) of Amsterdam-based GPs active in 2015–2020 attended one or both sessions (154 attended one of the two and 75 attended both), including 103 Session-II participants. In total, 101/103 (98%) participants of Session-II completed the questionnaire. Of these, 65 (64%) reported they had participated in both sessions and therefore could be included in analyses on the self-reported effect of the programme. Of these 65, eight (12%) participated in the interviews. Participant characteristics are described in Table 1.

**Table 1:** Characteristics of the participating Amsterdam-based GPs that completed the questionnaire, and participated in the interviews evaluating the effect of the educational intervention, 2017–2020

	**Questionnaire participants (*n* = 101)**	**Interview participants** **(*n* = 8)**
	**n (%)**	**n (%)**
Female sex	63 (62%)	3 (38%)
Age categories		
30–44 years	39 (37%)	0 (0%)
45–59 years	40 (40%)	6 (75%)
60 + years	22 (22%)	2 (25%)
Years work experience		
0–5 years	13 (13%)	0 (0%)
6–10 years	21 (21%)	1 (13%)
11–15 years	12 (12%)	0 (0%)
> 15 years	54 (54%)	7 (88%)
missing	1 (1%)	n/a
No. of days working per week		
< 3 days	5 (5%)	n/a
3–4 days	84 (84%)	
5 days (full-time)	11 (11%)	
missing	1 (1%)	n/a
Est. no. of PLHIV in the practice		
< 5 PLHIV	7 (7%)	0 (0%)
5–10 PLHIV	41 (41%)	5 (63%)
11–25 PLHIV	27 (27%)	1 (13%)
> 25 PLHIV	9 (9%)	2 (25%)
Don’t know	14 (14%)	0 (0%)
missing	3 (3%)	n/a
Participated in the first session	65 (64%)	8 (100%)
Additional HIV/STI related activities	n/a	3 (38%)

*GP* general practitioner, *PLHIV* people living with HIV, *STI* sexually transmitted infection

### Questionnaire-reported effect of the programme

Forty-five participants (69%) reported that Session-I provided eye-openers on HIV/STI testing. Ten eye-opener themes were identified, including becoming motivated to offer more extragenital STI tests (i.e. oropharyngeal or anorectal chlamydia and gonorrhoea testing), to improve STI testing based on a proper risk-assessment and the GPs’ guideline for STI consultations, to offer HIV testing more proactively and gaining awareness on HIV indicator conditions (Table 2). Forty-seven (72%) GPs reported the programme had changed their HIV/STI testing behaviour. Forty-two elaborated on these changes, and seven themes were identified, including improved STI consultations and adherence to the guidelines, more proactive HIV testing or -offering, and more extragenital STI testing when indicated (Table 3).

**Table 2:** Identified themes from the 45 Amsterdam GPs that reported Session-I provided eye-openers in questionnaires evaluating the effect of the educational intervention, 2017–2020

Theme
Motivation for more proactive extragenital STI testing (including oropharyngeal and anorectal)
Motivation to improve STI testing based on risk assessment and the guidelines for STI testing
Motivation for more proactive HIV testing
Awareness of HIV indicator conditions
Motivation for more HIV/STI testing in general
Awareness of other STI (syphilis, hepatitis C, *Mycoplasma genitalium*)
Awareness of the (undiagnosed) HIV prevalence
Awareness of the clinical symptoms of acute HIV infection
Less HIV test ordering in low-risk populations
Awareness that too little HIV/STI testing is being done

*GP* general practitioner, *Session-I* First session of the educational intervention, *STI* sexually transmitted infection

**Table 3:** Identified themes from the 42 Amsterdam GPs that reported how attending Session-I changed their HIV/STI testing behaviour in questionnaires evaluating the effect of the educational intervention, 2017–2020

Theme
Improved STI consultation; better history taking, following the guidelines
More extragenital STI testing when indicated
More proactive HIV testing or addressing HIV
More HIV/STI testing in general
More hepatitis C testing
More indicator condition-guided HIV testing
Started prescribing pre-exposure prophylaxis for HIV

*GP* general practitioner, *Session-I* First session of the educational intervention, *STI* sexually transmitted infection

The percentages of GPs who reported increased testing frequency for chlamydia, gonorrhoea and HIV were 27% (16/60), 23% (14/60), and 54% (31/58), respectively. The percentages who reported no change in testing for chlamydia, gonorrhoea and HIV were 73% (44/60), 50% (30/60), and 43% (25/58), respectively. The percentages who reported decreased testing for chlamydia, gonorrhoea and HIV were 0% (0/60), 27% (16/60) and 4% (2/58), respectively. Of the 24 GPs who reported no change in HIV testing, three made additional comments. One GP stated they offered HIV testing more frequently, but it was regularly refused by patients due to financial barriers. One GP stated they offered HIV testing only at the patients’ request. Another GP reported that despite their intention to increase HIV testing, they still did not test for HIV very often due to lack of time.

Sixty-seven GPs elaborated on when they would test their patient for the ‘Big 5’ (i.e. chlamydia, gonorrhoea, HIV, hepatitis B, and syphilis; the STIs recommended to test for in patients with significant STI risk [6]), and six themes were identified. The GP’s risk assessment, the patient’s request, a positive HIV/STI test result, the cost of STI tests, whether the patient attends the SHC and routine practices were identified factors (Supplementary Table 3).

### Implementation of quality improvement plans

GPs formulated up to five quality improvement plans for HIV/STI testing and counselling in their practice at the end of Session-I. GPs reported after Session-II that of these, 82% (139/169) were reportedly partially or completely implemented. By theme, reported implementation was highest among plans to improve extragenital testing and STI consultations in general (Supplementary Table 4). Implementation was lowest among plans to improve HIV testing and counselling, especially among GPs who had planned to inform patients about HIV testing on waiting room screens and those who had planned to offer HIV testing during routine health-checks. Seventeen GPs provided additional commentary and five themes were identified. Fear of worrying patients when providing information about HIV on waiting room screens, language barriers, rarely encountering HIV indicator conditions, becoming less proactive over time, and financial barriers were mentioned as barriers to implementation.

Ninety-one GPs (90%) completed an open question on further improvement plans for HIV/STI testing after attending Session-II. More focus on HIV, more frequent offering of HIV and extragenital STI testing, and further improving HIV/STI consultations in general, were among the themes that emerged. Additionally, 25% reported planning to start or to expand their prescribing of pre-exposure prophylaxis for HIV (PrEP) (Table 4).

**Table 4:** Identified themes from the 91 Amsterdam GPs who reported on what they planned to improve upon further after attending the second and final session of this programme in questionnaires evaluating the effect of the educational intervention, 2017–2020

Theme
More HIV testing or focus on HIV during consultations
More extragenital chlamydia/gonorrhoea testing including anorectal testing
Further improve HIV/STI testing and consultations in general
Start/expand prescribing of pre-exposure prophylaxis
More indicator condition-guided testing for HIV (including in case of another STI)
More testing or focus on hepatitis B/C during consultations
Improved *Mycoplasma genitalium* testing strategies (i.e. usually less testing)
More retesting for chlamydia after treatment for a chlamydia-infection

*GP* General practitioner, *SHC* sexual health centre

### Effects of the programme as reported by interviewees

From the interviews, fifteen themes on self-reported changes in HIV/STI testing following the intervention were identified. Some GPs reported less frequent HIV testing in low-risk patients, while others reported more frequent HIV testing, even in low-risk patients:

*Some patients that definitely have a low risk I now test less. Sometimes people want HIV testing done themselves, then it’s fine, but I’m less proactive (Female, 50 years, 17 years work-experience).*

*I was already proactive, but really exclusively in key groups and now I think, I should also test the low-risk groups. You kind of want to screen all of Amsterdam (Female, 54 years, 25 years work-experience).*

GPs reported being more alert and more proactive in HIV testing, and being more alert on HIV indicator conditions:

*I diagnosed someone with HIV recently. He had very severe eczema, one of those indicator conditions. You recognise this faster now (Female, 50 years, 17 years work-experience).*

Additionally, GPs reported that they became more motivated to increase HIV testing in MSM and (undocumented) migrants, and to increase awareness on HIV and HIV testing among their patient population.

Other themes on self-reported changes in HIV/STI testing that emerged were gaining more skills on how to discuss HIV testing and sexual health including sexual behaviour to determine the need for extragenital STI testing, and more awareness on extragenital STI. Finally, GPs reported having gained more knowledge on indications for STI testing.

### Interviewees’ reflection on trends in HIV testing

**Fig. 1:**
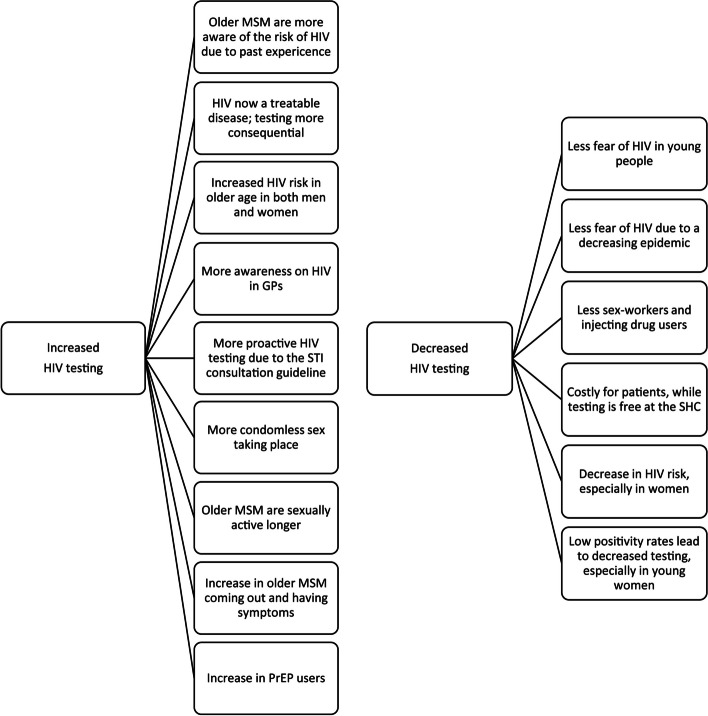
Themes identified in the interviews with eight GPs in Amsterdam regarding their reflections on trends in HIV testing, 2020. GP: general practitioner. MSM: men who have sex with men. PrEP: Pre-exposure prophylaxis. SHC: sexual health centre. STI: sexually transmitted infection

Fifteen themes regarding reflections on trends in HIV testing by GPs in Amsterdam were identified (Fig. 1). Reflections on the initial decrease in HIV testing included a declining prevalence, less perceived risk of HIV and financial barriers. Reflections on the increase in HIV testing from 2015 onward included increased condomless sex, lower threshold for HIV testing due to HIV becoming a treatable, chronic condition, HIV awareness campaigns, and GPs prescribing PrEP.

### Barriers and facilitators

Twenty-seven themes on persistent barriers and facilitators for HIV/STI testing were identified from the interviews and divided into barriers and facilitators at the patient-level (10), provider-level (8) and system-level (10) (Supplementary Table 5).

#### Patient-level

Participants mentioned perceiving patients’ fearful response to offering an HIV test as a barrier:

*I used to test very proactively, but I’m less on top of it now. But that’s also because people get very frightened when I bring it up. Sometimes I’ll just let them mull it over for a while (Female, 52 years, 27 years work-experience).*

Patient-level facilitators included easy discussion of HIV testing with key groups including MSM, while the fact that sexuality and homosexuality are taboo in some cultures was perceived as a patient-level barrier. Symptoms and HIV indicator conditions were frequently mentioned facilitators for testing:

*When someone has non-specific symptoms, such as weight loss or malaise, then at some point you think about who is in front of you, could it be an HIV infection? (Male, 53 years, 18 years work-experience)*

#### Provider-level

Provider-level barriers included lack of training on sexual health in the GP vocational training programme, GPs sticking to old patterns in testing strategies, and GPs feeling less motivated to test for HIV due to decreasing HIV prevalence in the Netherlands.

#### System-level

Participants mentioned cost of testing as a system-level barrier, which sometimes lead to less frequent testing, or referral to the SHC or other free testing services. Two participants reported that the comprehensiveness of the STI consultation guideline posed a barrier to adherence:

*We are a group that pre-eminently works based on past experience. So naturally, training and guidelines are important, but the guidelines are so elaborate that you don’t know them by heart, and then experience is leading (Female, 52 years, 27 years work-experience).*

### Evaluation of the programme

**Fig. 2:**
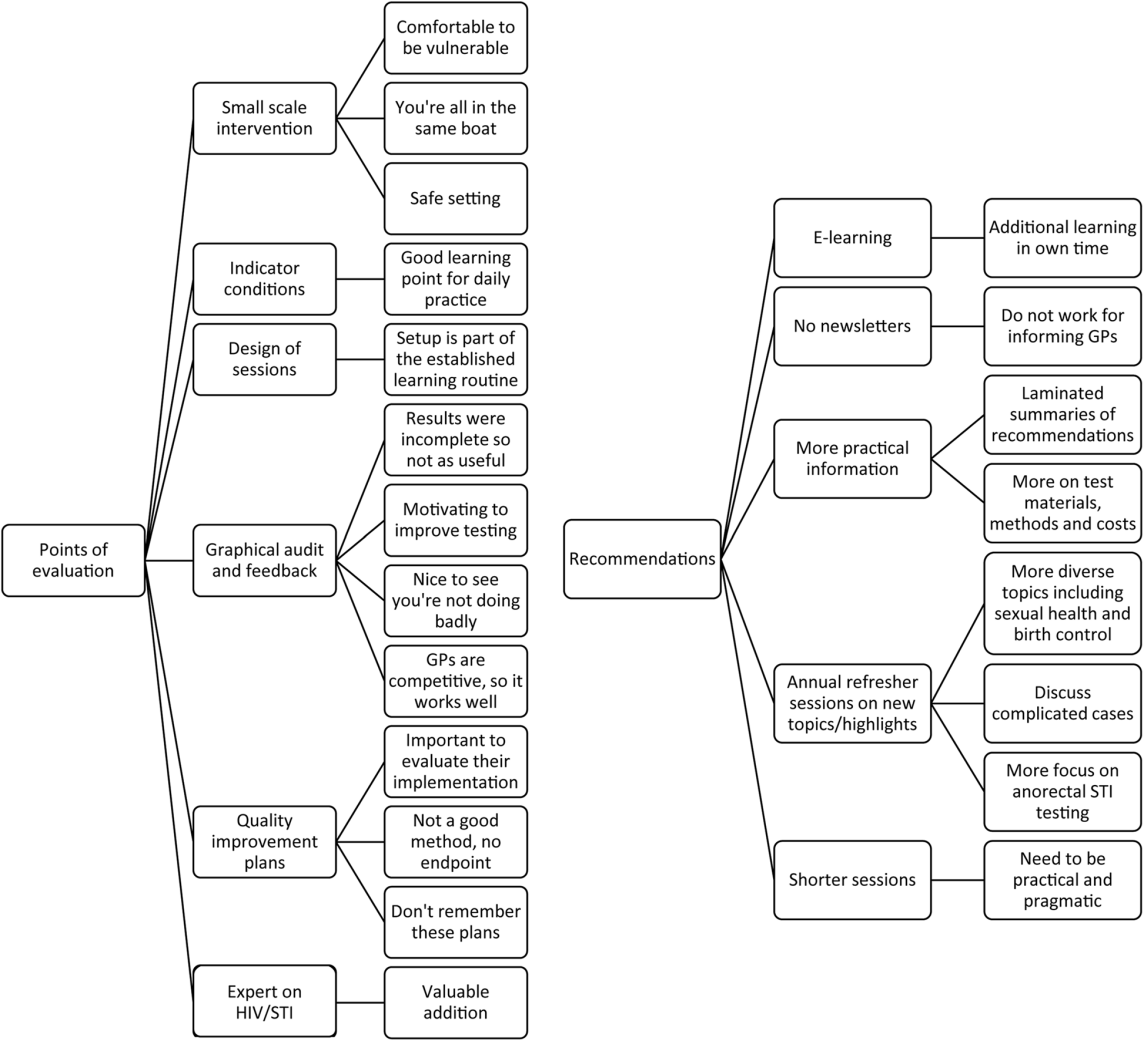
Themes identified in the interviews with eight GPs in Amsterdam on the evaluation and recommendations for improvement of the educational intervention, 2020. GP: general practitioner. STI: sexually transmitted infection

In the questionnaire, the programme received a mean grade of 8.4 (SD 0.7, range 7–10) on a 10-point scale. Thirty-six GPs (36%) completed an open question on what could be improved, and eight themes were identified. Change in duration of the session, more practical sessions, receiving more detailed or more recent audit and feedback, discussing a wider range of sexual health-related topics and repeat sessions were themes that emerged (Supplementary Table 6). In an evaluation by interviewees, several strengths and recommendations for improvement for the programme were identified (Fig. 2). Strengths included using already established training structures and using competitive audit and feedback to motivate sustained improvement:

You usually already attend continued medical education sessions with the same group of GPs, so you are allowed to be bewildered by other participants’ testing strategies, and to ask awkward questions, and to be vulnerable. So I think that’s very important (Male, 60 years, 25 years work-experience).

The funny thing is, GPs, however big-mouthed they are, they’re always a bit afraid that they are underperforming. I have that too. But then we get our audit and feedback and then it turns out we’re not doing too bad at all. That’s really motivating to see (Female, 52 years, 27 years work-experience).

You really get a big mirror held up to your own testing behaviour. So I think it really lasts, because it’s more than a quick fix, so it would really work in the long run (Male, 40 years, 10 years work-experience).

The use of quality improvement plans received additional feedback:

*I think we have about fifty practice improvement plans in our practice currently. You have to be careful about all these plans that sort of hang around, they start and never finish. It’s better to ask the group what they need, or one or two real take home messages, and address those in follow-up sessions, then it can be really effective (Male, 53 years, 18 years work-experience).*

## Discussion

In our study, we aimed to gain a deeper understanding of whether and how the quality and perceptions of GPs’ HIV/STI testing behaviour changed by the programme. While analyses of laboratory data showed a 7% increase in HIV testing frequency among participating GPs [11], the results of this mixed-methods study suggest that self-reported knowledge, attitude and testing by participating GPs in Amsterdam changed more substantially.

More than two-thirds of participants reported that the programme provided eye-openers on HIV/STI testing, and that it changed their testing behaviour. Over half of participants reported to have increased their HIV testing frequency, while the rest reported no change in frequency, or even a decrease in HIV testing. Reported improvements in HIV/STI testing behaviour included improved STI consultations and improved adherence to the guidelines, including testing for HIV/STI less when it is not indicated. GPs also reported increased willingness to start prescribing PrEP. The interactive, small-scale design of the sessions and the use of audit and feedback were positively evaluated and helped establish intrinsic motivation to improve HIV/STI testing behaviour. Additionally, inclusion of repeat sessions in the programme made sustainable improvement more likely [15–17].

We identified several system-level barriers including financial barriers (i.e. cost of HIV testing by GPs is only covered by health insurances if the annual deductible has been reached), and the STI consultation guideline being perceived as too extensive to be useful. Some GPs reported not having implemented their quality improvement plan to discuss HIV testing more frequently or to inform patients about HIV testing on waiting room screens, because they feared their patients would worry. This shows that HIV-related stigma remains an important issue [12, 18, 19], obstructing optimal HIV testing in primary care. This study further confirmed previous findings that both GPs’ and patients’ perceived risk remains one of the most important motivators for HIV testing [20, 21]. A shrinking HIV epidemic was mentioned as a reason for GPs to test for HIV less proactively, especially among low-risk groups. As we are entering a new era in the HIV epidemic, i.e. ‘the last mile’ towards reaching (micro)elimination of HIV in the Netherlands, a decreasing HIV prevalence will likely decrease GPs’ and patients’ perceived risk of HIV, compromising appropriate testing behaviour in the future.

The discrepancy between the modest increase in HIV testing frequency assessed with laboratory data and the more considerable self-reported improvements in HIV/STI testing behaviour found in this study may also be an indication that participants report intention rather than actual behaviour (i.e. “wishful thinking”), or that they overestimate the quality of their own testing behaviour, which in practice may have not improved considerably. This has been reported in other studies, that showed that self-assessment of quality of delivered care by healthcare providers is not always accurate [22–24]. Conversely, some participants reported intentional decreases in HIV/STI testing among low-risk patients, which might also explain the described discrepancy. Increased testing frequency does not necessarily mean improved HIV testing, which is more complex to assess as it may depend on the patient’s risk-profile, their symptoms and/or diagnoses and any findings during physical or laboratory examination. Moreover, decreased testing in asymptomatic, low-risk patients is only justified after a thorough risk-assessment, and previous research has shown that GPs are often unaware of certain risk-factors [25].

In recent years, the role of primary care physicians in optimal HIV testing is increasingly recognised, leading to several intervention studies to improve testing among GPs [15, 16, 26–29]. These studies yielded mixed results, including considerable increases in testing [26], as well as no effect, or even decreases in testing after implementation [15, 30]. In most of these studies, sustainability or generalisability of the effect was compromised due to limitations including temporary financial incentives, lack of follow-up and lack of combination approaches. In our study, willingness to participate in the educational programme posed a challenge in its implementation, as illustrated by the fact that only a third of the participants attended both sessions. GPs in the Netherlands are currently heavily overburdened [31], and have a wide range of topics to choose from in attending CME sessions. Combined with a diminished sense of urgency due to the shrinking HIV epidemic, this may make proper attention for HIV testing challenging. Therefore, designing CME projects that are specifically focused on sustainability of quality improvement, by incorporating a combination of audit and feedback, repeat sessions, and IT solutions such as electronic prompts will be key moving forward.

### Strengths and limitations

An important strength of our study is the fact that we used multiple data sources to gain a deeper understanding of the effect of the intervention on testing behaviour than laboratory data alone would have. Our study has several limitations, the most important being reporting bias and recall bias; participants may have given socially desirable answers and may have overestimated the quality of their testing behaviour. As questionnaires were completed anonymously, we could not compare self-reported changes in testing from questionnaire data to laboratory data per GP. Finally, selection bias may have occurred during the interviews, as several participants were involved in additional HIV/STI related activities. However, we did achieve theoretical data saturation with the included interviews, mitigating selection bias as much as possible.

## Conclusions

The majority of GPs attending an educational intervention programme reported improved HIV/STI testing behaviour, but stigma, decreasing perceived risk and several structural barriers hamper sustained improvement.

### Supplementary Information

**Additional file 1: Supplementary Table 1.** Questionnaire for Amsterdam-based GPs that attended Session-II of the educational programme, to evaluate the effect and acceptance of the programme, 2017-2020. **Supplementary Table 2.** Interview topic guide for interviews with Amsterdam-based GPs that attended both sessions of the educational programme, to evaluate the effect and acceptance of the programme, 2020. **Supplementary Table 3.** Identified themes from the 67 Amsterdam-based GPs that elaborated on their reasons to test for the Big 5 in questionnaires evaluating the effect of the educational intervention, 2017-2020. **Supplementary Table 4.** Implementation of quality improvement plans by theme reported by Amsterdam-based GPs that attended both educational session in questionnaires evaluating the effect of the educational intervention, 2017-2020. **Supplementary Table 5.** Themes identified in the interviews with eight GPs in Amsterdam regarding patient level, provider level and system level barriers and facilitators for HIV/STI testing, 2020. **Supplementary Table 6.** Identified themes from the 36 Amsterdam GPs who reported on what could be improved in the programme in questionnaires evaluating the effect of the educational intervention, 2017-2020.

## Data Availability

All data generated or analysed during this study are included in this published article and its supplementary information files.

## Abbreviations

CME: Continued medical education
GP: General practitioner
H-TEAM: HIV Transmission Elimination in Amsterdam Initiative
MSM: Men who have sex with men
PLHIV: People living with HIV
PrEP: Pre-exposure prophylaxis for HIV
SHC: Sexual health centre
STI: Sexually transmitted infection
