# Supplementary material for: Understanding the effect of an educational intervention to optimize HIV testing strategies in primary care in Amsterdam – results of a mixed-methods study
Source: BMC Prim Care. 2023 Sep 30;24:201. doi: 10.1186/s12875-023-02161-y (PMC10541686; doi:10.1186/s12875-023-02161-y)
Supplement: Supplementary file 1 — Additional file 1: Supplementary Table 1. Questionnaire for Amsterdam-based GPs that attended Session-II of the educational programme, to evaluate the effect and acceptance of the programme, 2017-2020. Supplementary Table 2. Interview topic guide for interviews with Amsterdam-based GPs that attended both sessions of the educational programme, to evaluate the effect and acceptance of the programme, 2020. Supplementary Table 3. Identified themes from the 67 Amsterdam-based GPs that elaborated on their reasons to test for the Big 5 in questionnaires evaluating the effect of the educational intervention, 2017-2020. Supplementary Table 4. Implementation of quality improvement plans by theme reported by Amsterdam-based GPs that attended both educational session in questionnaires evaluating the effect of the educational intervention, 2017-2020. Supplementary Table 5. Themes identified in the interviews with eight GPs in Amsterdam regarding patient level, provider level and system level barriers and facilitators for HIV/STI testing, 2020. Supplementary Table 6. Identified themes from the 36 Amsterdam GPs who reported on what could be improved in the programme in questionnaires evaluating the effect of the educational intervention, 2017-2020. [file 12875_2023_2161_MOESM1_ESM.docx]

Supplementary online material to:

**Title:** Understanding the effect of an educational intervention to optimize HIV testing strategies in primary care in Amsterdam – Results of a Mixed-methods study.

**Running title:** Evaluation of intervention for HIV testing by GPs

**Authors:**

Saskia Bogers^1-3^, Pythia Nieuwkerk^4^, Nynke van Dijk^5,6^, Maarten Schim van der Loeff^1,7^, Suzanne Geerlings^1-3^ and Jan van Bergen^5,8^ on behalf of the HIV Transmission Elimination Amsterdam (H-TEAM) initiative

**Author affiliations:**

1 Amsterdam UMC location University of Amsterdam, Department of Internal Medicine, Meibergdreef 9, Amsterdam, The Netherlands

2 Amsterdam institute for Infection and Immunity, infectious diseases, Amsterdam, the Netherlands

3 Amsterdam Public Health research institute, Quality of care, Amsterdam, the Netherlands

4 Amsterdam UMC location University of Amsterdam, Department of Medical Psychology,

Meibergdreef 9, Amsterdam, The Netherlands

5 Amsterdam UMC location University of Amsterdam, Department of General Practice,

Meibergdreef 9, Amsterdam, The Netherlands

6 Amsterdam University of Applied Sciences, Faculty of Health, Center of Expertise Urban Vitality Amsterdam, The Netherlands

7 Department of Infectious Diseases, Public Health Service of Amsterdam, Amsterdam, the Netherlands.

8 STI AIDS Netherlands, Amsterdam, the Netherlands.

**Corresponding author:**

Saskia J. Bogers, MD

Amsterdam UMC-AMC, department of Internal Medicine

Room D3-226, Meibergdreef 9, 1105 AZ Amsterdam, The Netherlands

E-mail: [s.j.bogers@amsterdamumc.nl](mailto:s.j.bogers@amsterdamumc.nl)

Supplementary Table 1: Questionnaire for Amsterdam-based GPs that attended Session-II of the educational programme, to evaluate the effect and acceptance of the programme, 2017-2020.

| **1. Participant characteristics** | | |
| --- | --- | --- |
| Sex of participant | Male/Female |  |
| Age of participant | 20-24 years/25-29 years/30-34 years/35-39 years/40-44 years/45-49 years.50-54 years/55-59 years/60-64 years/≥65 years |  |
| Job description of participant | General practitioner / other, i.e.: |  |
| Number of years working as a general practitioner | GP in training/0-5 years/6-10 years/11-15 years/>15 years/ not a GP |  |
| Type of practice of participant | Single/Dual/Group/Health centre/other, i.e.: |  |
| Number of days per week working as a GP | *[Open question]* |  |
| Number of people living with HIV registered in the participant’s practice | <5 patients/5-10 patients/11-25 patients/>25 patients/Don’t know |  |
| **2a. Effect of the previous educational session** | | |
| 1. To what extent do you recall topics that were discussed during Session-I? | a. I don’t remember Session-I at all  b. I don’t remember much from Session-I  c. I remember a few topics that were discussed  d. I remember many topics that were discussed  e. I remember most topics that were discussed  f. I remember everything from Session-I  g. I did not attend Session-I 🡪 Skip to question 5 |  |
| 2. Did Session-I provide eye-openers on HIV/STI testing? | a. No/b. I don’t remember/c. Yes, i.e.: |  |
| 3. To what extent did the educational sessions change your HIV/STI testing behaviour? | a. I did not change my HIV/STI testing behaviour at all due to the educational sessions  b. I changed my HIV/STI testing behaviour somewhat due to the educational sessions, i.e.:  c. I changed my HIV/STI testing behaviour a lot due to the educational sessions, i.e.:  d. I don’t know |  |
| 4a. To what extent did you change your chlamydia testing behaviour? Please elaborate | a. Much less testing/b. Less testing/c. No change/d. More testing/e. Much more testing |  |
| 4b. To what extent did you change your gonorrhoea testing behaviour? Please elaborate | a. Much less testing/b. Less testing/c. No change/d. More testing/e. Much more testing |  |
| 4c. To what extent did you change your HIV testing behaviour? Please elaborate | a. Much less testing/b. Less testing/c. No change/d. More testing/e. Much more testing |  |
| **2b. Reported changes in HIV/STI testing in thirteen hypothetical clinical consultation situations**  ***(leave ‘before Session-I blank if you did not attend Session-I)*** | | |
| 5. I test a high-risk patient for the Big 5.  a. Before attending Session-I  b. After attending Session-I | 1. Never/2. Rarely/3. Regularly /4. Often/5. Always/6. Don’t know |  |
| 6a. When I perform an STI test I also perform an anorectal test in MSM  a. Before attending Session-I  b. After attending Session-I | 1. Never/2. Rarely/3. Regularly /4. Often/5. Always/6. Don’t know |  |
| 6b. When I perform an STI test I also perform an anorectal test in women with anorectal sexual contact  a. Before attending Session-I  b. After attending Session-I | 1. Never/2. Rarely/3. Regularly /4. Often/5. Always/6. Don’t know |  |
| 7. I advise a patient with chlamydia to do a repeat chlamydia test within 3-12 months  a. Before attending Session-I  b. After attending Session-I | 1. Never/2. Rarely/3. Regularly /4. Often/5. Always/6. Don’t know |  |
| 8. When I order a chlamydia test I also order a gonorrhoea test  a. Before attending Session-I  b. After attending Session-I | 1. Never/2. Rarely/3. Regularly /4. Often/5. Always/6. Don’t know |  |
| 9. I offer a patient with gonorrhoea an HIV test  a. Before attending Session-I  b. After attending Session-I | 1. Never/2. Rarely/3. Regularly /4. Often/5. Always/6. Don’t know |  |
| 10a. I offer all my patients an HIV test when I am performing a blood test  a. Before attending Session-I  b. After attending Session-I | 1. Never/2. Rarely/3. Regularly /4. Often/5. Always/6. Don’t know |  |
| 10b. I offer MSM an HIV test when I am performing a blood test  a. Before attending Session-I  b. After attending Session-I | 1. Never/2. Rarely/3. Regularly /4. Often/5. Always/6. Don’t know |  |
| 10c. I offer migrants from HIV endemic countries an HIV test when I am performing a blood test  a. Before attending Session-I  b. After attending Session-I | 1. Never/2. Rarely/3. Regularly /4. Often/5. Always/6. Don’t know |  |
| 11. I offer patients aged <60 years with herpes zoster (shingles) an HIV test  a. Before attending Session-I  b. After attending Session-I | 1. Never/2. Rarely/3. Regularly /4. Often/5. Always/6. Don’t know |  |
| 12. I offer patients with a hepatitis B infection an HIV test  a. Before attending Session-I  b. After attending Session-I | 1. Never/2. Rarely/3. Regularly /4. Often/5. Always/6. Don’t know |  |
| 13. I consider HIV infection in patients presenting with mononucleosis-like symptoms  a. Before attending Session-I  b. After attending Session-I | 1. Never/2. Rarely/3. Regularly /4. Often/5. Always/6. Don’t know |  |
| 14. I offer patients with unexplained symptoms such as chronic diarrhoea or weight loss an HIV test  a. Before attending Session-I  b. After attending Session-I | 1. Never/2. Rarely/3. Regularly /4. Often/5. Always/6. Don’t know |  |
| **3. Implementation of quality improvement plans for HIV/STI testing in practice**  **(During Session-I, your group established the below listed quality improvement plans. Please report how well they were implemented)** | | |
| *[Quality improvement plan 1]* | a. Not implemented/b. Partially implemented/c. Completely implemented  Explanation … |  |
| *[Quality improvement plan 2]* | a. Not implemented/b. Partially implemented/c. Completely implemented  Explanation … |  |
| *[Quality improvement plan 3]* | a. Not implemented/b. Partially implemented/c. Completely implemented  Explanation … |  |
| *[Quality improvement plan 4]* | a. Not implemented/b. Partially implemented/c. Completely implemented  Explanation … |  |
| **4. Evaluation of the educational programme** | | |
| 1. What did you gain from attending this educational programme? | *[Open question]* |  |
| 2. How would you grade the quality if this programme on a 10-point scale? | 1/2/3/4/5/6/7/8/9/10 |  |
| 3. How can the programme be improved in the future? | *[Open question]* |  |
| 4. Are you planning on making any (further) changes in your HIV/STI testing behaviour after attending this Session-II? If so, which? | *[Open question]* |  |

Big 5: chlamydia, gonorrhoea, HIV, hepatitis B, and syphilis. GP: general practitioner. MSM: men who have sex with men. STI: sexually transmitted infection

Supplementary Table 2: Interview topic guide for interviews with Amsterdam-based GPs that attended both sessions of the educational programme, to evaluate the effect and acceptance of the programme, 2020.

| Topic | Addressed questions |
| --- | --- |
| (0) Participant characteristics | - Age, sex, number of years working as a GP, type of practice, neighbourhood of practice, estimated number of patients with HIV in practice, additional activities related to HIV/STI care |
| (1) HIV/STI testing behaviour, barriers and facilitators | - How would you describe your HIV/STI testing behaviour?  - Which barriers and facilitators to HIV/STI testing do you experience?  - Which other factors have influenced your HIV/STI testing behaviour over time? |
| (2) Changes in HIV/STI testing behaviour following the educational intervention | - How has your HIV/STI testing behaviour changed following the educational intervention?  - Has your provider-initiated HIV/STI testing behaviour changed (how)?  - Are there patient groups that you test more often or less often following the intervention (which, why)?  - Has the educational intervention changed your perceived barriers and facilitators to HIV/STI testing (how)?  - Are any perceived changes likely to be sustainable? |
| (3) Reflection on observed changes in HIV/STI testing trends by GPs in Amsterdam using graphs based on laboratory data | - How would you explain the observed changed in HIV/STI testing and positivity percentages by Amsterdam GPs over time? And when stratified by sex and age categories? (graphs for trends in HIV, chlamydia and gonorrhoea – both genitourinary and anorectal – testing and positivity are discussed)  - Which influencing factors could have played a role in these changes (how)? Could the intervention have played a role (how)? |
| (4) Evaluation of the educational intervention programme | - How would you evaluate the educational intervention programme?  - Are there specific topics that you remember most from the educational intervention?  - What was most useful in the design? What was least useful? What did you miss?  - How would you evaluate the use of graphical audit and feedback in the sessions?  - How would you evaluate the use of quality improvement plans in the sessions?  - How would you evaluate the attendance of an expert in the field of HIV/STI in primary care in the sessions?  - How would you evaluate the sessions being held in a small group of peers/colleagues?  - How would you improve the sessions? Do you have any other advice/tips? |

GP: general practitioner. STI: sexually transmitted infection

Supplementary Table 3: Identified themes from the 67 Amsterdam-based GPs that elaborated on their reasons to test for the Big 5 in questionnaires evaluating the effect of the educational intervention, 2017-2020.

| **Theme** | **n (%*)** |
| --- | --- |
| My risk assessment of the patient is a factor in deciding to test for the Big 5 or not | 41 (61%) |
| The patient’s request or choice is a factor in deciding to test for the Big 5 or not | 23 (34%) |
| A positive HIV/STI result or symptoms are a factor in deciding to test for the Big 5 or not | 13 (19%) |
| The costs of STI tests are a factor in deciding to test for the Big 5 or not | 10 (15%) |
| Whether the patient attends the SHC is a factor in deciding to test for the Big 5 or not | 4 (6%) |
| I routinely test all patients for the Big 5 when I perform STI testing | 3 (5%) |

* Percentages add up to >100% as GPs could provide as many reasons to test for the Big 5 as they wanted. Big 5: chlamydia, gonorrhoea, HIV, hepatitis B, and syphilis. GP: general practitioner. STI: sexually transmitted infection. SHC: sexual health centre.

Supplementary Table 4: Implementation of quality improvement plans by theme reported by Amsterdam-based GPs that attended both educational session in questionnaires evaluating the effect of the educational intervention, 2017-2020.

|  |  | Implementation of plans: | | |  |
| --- | --- | --- | --- | --- | --- |
| Quality improvement plan | No. of respondents | Not  n (%) | Partially  n (%) | Completely  n (%) |  |
| Theme 1: Improved STI consultation and testing | | | | | |
| 1- Improved history-taking during STI consultations | 20 | 1 (5%) | 9 (45%) | 10 (50%) |  |
| 2- Always perform STI test according to the STI consultation guideline | 15 | 0 (0%) | 11 (73%) | 4 (27%) |  |
| 3- No more over-the-counter STI testing, always perform a consultation | 9 | 1 (11%) | 4 (44%) | 4 (44%) |  |
| 4- Only test for chlamydia in patients at low-risk for STI | 8 | 1 (13%) | 0 (0%) | 7 (88%) |  |
| 5- Instruct assistance/create awareness on appropriate STI testing | 3 | 2 (67%) | 1 (33%) | 0 (0%) |  |
| 6- Repeat chlamydia testing in patients with chlamydia after 6 months | 2 | 1 (50%) | 0 (0%) | 1 (50%) |  |
| Total theme 1: Improved STI consultation and testing | 57 | 6 (11%) | 25 (44%) | 26 (46%) |  |
| Theme 2: Improved HIV consultation and testing | | | | | |
| 7- Offer HIV testing to risk-groups (incl. migrants from endemic countries) | 26 | 5 (19%) | 19 (73%) | 2 (8%) |  |
| 8- Offer HIV testing more proactively | 11 | 2 (18%) | 6 (55%) | 3 (27%) |  |
| 9- Offer HIV testing in the case of HIV indicator conditions | 10 | 2 (20%) | 5 (50%) | 3 (30%) |  |
| 10- Inform patients about HIV (testing) on waiting room screens | 8 | 5 (63%) | 0 (0%) | 3 (38%) |  |
| 11- Offer HIV testing during routine ‘health-checks’ | 3 | 1 (33%) | 2 (67%) | 0 (0%) |  |
| 12- Offer HIV testing to new patients during intake | 3 | 0 (0%) | 0 (0%) | 3 (100%) |  |
| Total theme 2: Improved HIV consultation and testing | 61 | 15 (25%) | 32 (53%) | 14 (23%) |  |
| Theme 3: Continued learning on HIV/STI | | | | | |
| 13- Repeat this learning session in 1-2 years | 14 | 2 (14%) | 4 (29%) | 8 (57%) |  |
| 14- Send out a quarter-year newsletter on appropriate HIV/STI testing | 7 | 2 (29%) | 4 (57%) | 1 (14%) |  |
| 15- Evaluate our quality improvements in one year | 3 | 2 (67%) | 1 (33%) | 0 (0%) |  |
| Total theme 3: Continued learning on HIV/STI | 24 | 6 (25%) | 9 (38%) | 9 (38%) |  |
| Theme 4: Improved extragenital testing | | | | | |
| 16- Improved extragenital testing when indicated | 22 | 2 (9%) | 13 (59%) | 7 (32%) |  |
| 17- Always perform extragenital chlamydia/gonorrhoea testing in MSM | 5 | 1 (20%) | 1 (20%) | 3 (60%) |  |
| Total theme 4: Improved extragenital testing | 27 | 3 (11%) | 14 (52%) | 10 (37%) |  |

GP: general practitioner. STI: sexually transmitted infection. MSM: men who have sex with men.

Supplementary Table 5: Themes identified in the interviews with eight GPs in Amsterdam regarding patient level, provider level and system level barriers and facilitators for HIV/STI testing, 2020.

| **Barriers** | **Facilitators** |
| --- | --- |
| **Patient level** | |
| - It worries the patient to bring it up | - Easy to discuss with MSM |
| - I do not test in the case of a low HIV risk heterosexual patient | - Key groups do not mind getting tested regularly |
| - Discussing (homo)sexuality is taboo in some cultures | - Additional STI testing in case of a positive test result |
| - Patients never ask for HIV testing themselves, I have to bring it up | - Amsterdam has a higher concentration of key groups |
|  | - HIV/STI testing depends on the patients risk profile and request |
|  | - HIV/STI testing depends on symptoms or HIV indicator conditions |
| **Provider level** | |
| - Discussing STI testing with patients you are not familiar with is harder | - I routinely test chlamydia and gonorrhea together |
| - Discussing STI testing with patients you are very familiar with is harder | - I am MSM, so I have a low threshold to offer extragenital testing |
| - Too little training on sexual health consultations in medical education |  |
| - Decreasing HIV epidemic makes testing less rewarding |  |
| - Old patterns of HIV stigma in older GPs |  |
| - I regularly forget discussing extragenital sexual contact |  |
| **System level** | |
| - Communication and collaboration with the SHC is suboptimal | - Only selected key groups have access to the SHC |
| - Chlamydia and gonorrhea are now separate on the order form | - Publications in medical journals for primary care improve testing |
| - Costs of testing influences test-ordering | - Informed consent is no longer needed for HIV testing |
| - Sex workers are not in the picture due to restrictive regulations | - Increased testing due to increased PrEP prescribing in primary care |
| - Other testing services for key groups such as online services and SHC are (more) popular |  |
| - STI guideline is too elaborate and comprehensive to be useful |  |

GP: general practitioner. SHC: sexual health centre. STI: sexually transmitted infection. MSM: men who have sex with men. PrEP: pre-exposure prophylaxis for HIV.

Supplementary Table 6: Identified themes from the 36 Amsterdam GPs who reported on what could be improved in the programme in questionnaires evaluating the effect of the educational intervention, 2017-2020.

| **Theme** | **n (%*)** |
| --- | --- |
| More time/longer sessions | 9 (25%) |
| Audit and feedback per GP practice, not per individual GP | 8 (22%) |
| Shorter/more practical/succinct sessions | 6 (17%) |
| Additional specific audit and feedback (e.g. per number of patients, as percentage, by sex) | 5 (14%) |
| Discussion of additional topics (e.g. syphilis, HPV, SHC data) | 4 (11%) |
| More recent audit and feedback | 4 (11%) |
| Periodic repeat sessions (after the two sessions of this programme) | 2 (6%) |
| More interactive discussion of clinical cases | 1 (3%) |

* Percentages add up to >100% as GPs could provide multiple examples of what could be improved. GP: general practitioner. HPV: human papillomavirus. SHC: sexual health centre.
